# Adult ADHD Patients in Community Mental Health Teams – an Unmet Need

**DOI:** 10.1192/bjo.2022.376

**Published:** 2022-06-20

**Authors:** Ayat Agabani, Lenny Cornwall, Sumeet Gupta, Helen Oatway, Hayli Ingram

**Affiliations:** 1 Health Education North East, Newcastle, United Kingdom; 2 Tees Esk and Wear Valley NHS Foundation Trust, Teesside, United Kingdom; 3 Tees Esk and Wear Valley NHS Foundation Trust, Harrogate, United Kingdom

## Abstract

**Aims:**

This Service Survey is a part of a Quality improvement project which aims to :1- To assess the extent of the problem regarding accessing Adult ADHD assessment and treatment by getting the views of clinicians.2- Evaluate negative impact on care coordinators of the delay in accessing timely and effective diagnosis and treatment of ADHD; This will: a-Increase understanding of the care needed by this patient group. b-Clarify current practice and any difficulties staff face in condition management when diagnosis not confirmed i.e. outline training needs. c-Determine if waiting time for diagnosis results in iatrogenic harm (deterioration driven by ‘unmet need’). 3-Inform the development of an alternative pathway of care; thus: a-Reduce inequality of healthcare access for those with this neurodevelopmental condition. b-Reduce stigma. c-Improve service user health and well-being. d-Support families and carers. e-Reduce social costs to individual and community. f-Support community staff and increase knowledge and effectiveness.

**Methods:**

Methods of the service survey part:

1. Service survey: Sent to 21 consultants who are working in Adult CMHT.
2. Service Satisfaction survey for all of the Redcar & Cleveland Affective disorder team's clinical staff members (18).

**Results: Consultants Service Survey:**

11 consultants responded out of 21 (52%)

Approximate number of the diagnosed ADHD patients / team varied between 7–80 patients.

Wait time for an ADHD assessment varied between 12 -30 months.

Number of patients/ team waiting for assessments by the specialist team 2- 27 patients.

50% of the consultants reported significant delays between referral to the services and initiation of treatment 6–36 months.

All consultants reported commencing treatment of ADHD, if a patient already had the diagnosis.

9/11 (82%) consultants reported making the initial diagnosis and treating ADHD patients in CMHTs. However, all consultant reported the need for further training in assessment and management of ADHD patients.

6/11 (55%) consultants stated that ADHD patients should be managed in CMHTs provided they are care coordinated by another clinician.

**Clinical Staff Satisfaction Survey:**

All 3 staffs responded out of 18 staff, reported un satisfaction with the current service provision.

**Conclusion:**

1. The current service model is not able to meet the increasing demand for the services and leading to significant delay in accessing appropriate treatment.
2. There is a need to improve competencies of community mental health teams to manage these patients.
3. This survey will be used to model a new care pathway.

